# Neonatal Exendin-4 Reduces Growth, Fat Deposition and Glucose Tolerance during Treatment in the Intrauterine Growth-Restricted Lamb

**DOI:** 10.1371/journal.pone.0056553

**Published:** 2013-02-12

**Authors:** Kathryn L. Gatford, Siti A. Sulaiman, Saidatul N. B. Mohammad, Miles J. De Blasio, M. Lyn Harland, Rebecca A. Simmons, Julie A. Owens

**Affiliations:** 1 Robinson Institute, University of Adelaide, Adelaide, South Australia, Australia; 2 School of Paediatrics and Reproductive Health, University of Adelaide, Adelaide, South Australia, Australia; 3 Department of Pediatrics, Perelman School of Medicine, University of Pennsylvania, Philadelphia, Pennsylvania, United States of America; CRCHUM-Montreal Diabetes Research Center, Canada

## Abstract

**Background:**

IUGR increases the risk of type 2 diabetes mellitus (T2DM) in later life, due to reduced insulin sensitivity and impaired adaptation of insulin secretion. In IUGR rats, development of T2DM can be prevented by neonatal administration of the GLP-1 analogue exendin-4. We therefore investigated effects of neonatal exendin-4 administration on insulin action and β-cell mass and function in the IUGR neonate in the sheep, a species with a more developed pancreas at birth.

**Methods:**

Twin IUGR lambs were injected s.c. daily with vehicle (IUGR+Veh, n = 8) or exendin-4 (1 nmol.kg^-1^, IUGR+Ex-4, n = 8), and singleton control lambs were injected with vehicle (CON, n = 7), from d 1 to 16 of age. Glucose-stimulated insulin secretion and insulin sensitivity were measured *in vivo* during treatment (d 12–14). Body composition, β-cell mass and *in vitro* insulin secretion of isolated pancreatic islets were measured at d 16.

**Principal Findings:**

IUGR+Veh did not alter *in vivo* insulin secretion or insulin sensitivity or β-cell mass, but increased glucose-stimulated insulin secretion *in vitro*. Exendin-4 treatment of the IUGR lamb impaired glucose tolerance *in vivo*, reflecting reduced insulin sensitivity, and normalised glucose-stimulated insulin secretion *in vitro*. Exendin-4 also reduced neonatal growth and visceral fat accumulation in IUGR lambs, known risk factors for later T2DM.

**Conclusions:**

Neonatal exendin-4 induces changes in IUGR lambs that might improve later insulin action. Whether these effects of exendin-4 lead to improved insulin action in adult life after IUGR in the sheep, as in the PR rat, requires further investigation.

## Introduction

Small size at birth or intrauterine growth restriction (IUGR) consistently predicts increased risk of type 2 diabetes mellitus (T2DM) in human studies [1], [2], including independently of gestation length [3]. This relationship is consistent and significant, with ∼18% of the lifetime risk of T2DM accounted for by poor growth before birth [4]. Impaired insulin sensitivity and inadequate insulin secretion are each implicated as contributing to this increased risk of T2DM in the IUGR human [1], [5], [6], [7].

Poor fetal growth commonly reflects restricted fetal supply of oxygen and nutrients due to impaired placental growth and/or function [8]. In the sheep, surgically-induced restriction of placental growth (PR) from before mating, and small size at birth, increase insulin sensitivity in early neonatal life in association with catch-up growth and increased fat deposition [9], [10]. PR nevertheless impairs glucose-stimulated insulin disposition before weaning at 1 month of age, and this progresses to impaired insulin sensitivity and blunted basal and glucose-stimulated insulin disposition in young adult males at 1 year of age [11], [12]. Impaired β-cell function is the primary cause of this inadequate insulin secretion, which occurs despite increases in β-cell mass in 1-year-old males [12]. Similarly, PR late in pregnancy in rats produces progeny with normal circulating glucose and insulin levels at 1 week of age, but mild fasting hyperglycemia and hyperinsulinemia at 7–10 weeks and frank diabetes by 26 weeks [13], [14]. Impaired β-cell function with later reduction in β-cell mass is also implicated in decreased insulin secretion in the PR rat postnatally [13], [14]. Excitingly, administration of the GLP-1 analogue exendin-4 to neonatal PR rats normalised subsequent β-cell mass and insulin secretion and prevented later development of T2DM [15]. Prevention of T2DM by neonatal exendin-4 treatment in PR rats is at least partially due to induction and normalisation of expression of the transcription factor *Pdx-1* [15], [16], which regulates β-cell function as well as adaptive increases in β-cell mass [17], [18], and is epigenetically down-regulated in PR rat progeny [19].

The timing of pancreatic development and maturation of β-cell function, and therefore developmental stages of exposure to IUGR and neonatal interventions, differs between species. In humans and sheep, most pancreatic development takes place before birth, with β-cells present by 0.25 gestation, islets present in mid-gestation and substantial remodelling to a mature endocrine pancreas by near term [20], [21], [22], [23]. In both species, β-cell function is present and matures from mid-gestation onwards [24], [25], [26], [27]. This functional maturation in humans and sheep may be driven in part by their pre-partum surge in cortisol. In contrast, rodents undergo later development of β-cells than sheep or humans, with β-cells first appearing in late gestation (0.6) and pancreatic remodelling at ∼10-17 d postnatal age [28], [29], [30]. Neonatal surges in corticosterone and β-cell maturation in rodents are marked by increased expression of key molecular determinants of glucose-induced insulin secretion coupling [31] and mitochondrial enzymes of the NADH shuttle, essential for stimulation of insulin secretion by oxidative metabolism [32]. Exendin-4 may in part be effective in preventing PR programming of reduced β-cell mass and function in rodents, because it occurs before and during such maturation. In the present study, we have therefore treated neonatal IUGR sheep with exendin-4 and assessed whether it is able to induce changes in growth, insulin action and β-cell mass and function after IUGR in a species in which the pancreas undergoes most maturation before birth.

## Materials and Methods

### Ethics statement

All procedures in this study were approved by the University of Adelaide Animal Experimentation and Ethics Committee (approval M-84-2007) and complied with the Australian code of practice for the care and use of animals for scientific purposes [33].

### Animal, treatments and surgery

Australian Merino ewes underwent a timed-mating program, and pregnancies were confirmed by ultrasound scanning at ∼60 d gestational age (term∼150 d). Delivery occurred naturally at term and the lambs were housed in floor pens with their mothers throughout the study and allowed to suckle freely, with access to their mother's feed and water, except during experimental protocols as described below. Natural twinning was used to induce IUGR. Sibling twin lambs were injected with vehicle (0.5% methanol in 0.9% saline s.c., IUGR+Veh) or exendin-4 (1 nmol.kg^−1^ s.c., IUGR+Ex-4, n = 8), with the first twin pair randomly allocated to treatments and then the heavier and lighter birth weight twin alternately allocated in order to balance birth weights between the two treatments. Exendin-4 (Bachem, Buberndorf, Germany) was prepared as a 5 nM stock in 0.5% methanol and 0.9% saline, and stored at −20°C in single use aliquots, which were thawed immediately prior to injection. Singleton lambs were injected daily with vehicle (CON, n = 7). All lambs (singletons and twins) were supplemented with whey protein (Resource Beneprotein instant protein powder, Nestle, Australia) given orally in two equal feeds (at 0900–1000 h and 1600–1700 h), commencing at 1.25 g.kg^−1^.d^−1^ on d 4 and increasing to 5 g.kg^−1^.d^−1^ on and after d 7. Feeding this supplement during this period of maximal catch-up growth in IUGR lambs [10] was intended to minimise the potential for limitation of neonatal growth by milk availability in twins [34] by providing ∼25% of the protein expected to be available through milk, and allowing lambs to self-regulate their milk intake to appetite.

On d 4, catheters were inserted into the lamb's femoral artery and vein under general anaesthesia, induced and maintained by fluothane inhalation anaesthetic, as described previously [10]. Basal blood samples were collected from arterial catheters every second morning before supplement feeding. Lambs were weighed at birth and then every 2 d throughout the study. Lamb size was measured at birth and then every 4 d, and absolute (AGR) and fractional (FGR) growth rates from birth to d 16 fitted by linear regression [10].

### *In vivo* measures of insulin secretion, sensitivity, and action

Glucose tolerance and glucose-stimulated insulin secretion were measured during an intravenous glucose tolerance test (IVGTT) at d 14, and indices of glucose tolerance and insulin secretion calculated as described previously [10], [11], [35]. The whole body insulin sensitivity of glucose metabolism was measured by hyperinsulinemic euglycemic clamp at d 12 [35]. Insulin sensitivityglucose, the metabolic clearance rate (MCR) of insulin, basal and maximal post-hepatic insulin delivery rates, and basal and maximal insulin disposition indices (IDI) were calculated as described previously [35].

### Analysis of plasma insulin and metabolites

Plasma insulin concentrations were measured in duplicate by a double antibody, solid phase radioimmunoassay using a commercially available kit (Human insulin-specific RIA, HI-14K, Linco Research Inc., St Charles, MO, USA), which has 100% cross-reactivity with ovine insulin. The intra-assay coefficients of variation (CV) for the insulin assay were 7.2% and 5.3%, and inter-assay CV were 7.0% and 19.6% for QC samples containing 9.9 and 35.9 mU.L−1 insulin respectively (n = 10 assays). Plasma glucose concentrations were measured by colorimetric enzymatic analysis on a Hitachi 912 automated metabolic analyser using Roche/Hitachi Glucose/HK kits (Roche Diagnostics GmbH, Mannheim, Germany).

### Post-mortem

Lambs were euthanized by overdose of sodium pentobarbitone at d 16. Organs (liver, kidneys, lungs, heart), muscles (semitendinosus, gastrocnemius, soleus, tibialis, extensor digitorum longus, biceps femoris, vastus lateralis, biceps), and dissectable fat depots (left and right perirenal fat, left and right retroperitoneal fat and omental fat) were dissected and weighed for each lamb. Dissected muscle and visceral fat weights were calculated as the sum of weights of these muscles and fat depots, respectively.

### Pancreas and islet isolation and immunostaining and morphometric analysis

Each pancreas was rapidly dissected and weighed. Representative mixed aliquots were fixed for 48 h in 4% paraformaldehyde before embedding in paraffin wax. One section per block was immunostained to detect insulin-positive cells, and morphometric analysis of β-cells was performed as described previously, in 20 fields of view per sheep selected by random-systematic sampling [12]. Measures of *in vivo* β-cell function were calculated by dividing total, 1^st^ phase and 2^nd^ phase glucose-stimulated insulin secretion and basal and maximal IDI by β-cell mass. Pancreatic islets were obtained by collagenase digestion of pancreas at 35°C for 40 min, washing and handpicking of islets >100 µm in diameter, with purity confirmed by immunostaining of aliquots as previously described [36]. Islet aliquots were cultured overnight at 37°C in 95% O_2_/5% CO_2_ in RPMI 1640 media (Sigma Aldrich, Sydney, Australia).

### *In vitro* β-cell secretion and responses

Static islet incubation and experiments were performed as previously described [36]. Briefly, for each animal and incubation condition, triplicate preparations of 10 islets were handpicked into 1.5 mL tubes. Static incubations were performed at 37°C for 1 hr in KRB/BSA/Forskolin media containing 0, 1.1, 11.1 mM glucose, or 15 mM KCl, or 11.1 glucose plus 5 mM Lysine, 11.1 glucose plus 5 mM Arginine, 1.1 glucose plus 10 mM Leucine, 11.1 glucose plus 10µM Epinephrine, or at 0°C for 1 hr in KRB/BSA/Forskolin media containing 11.1 glucose. Islets were then centrifuged, supernatant collected for insulin analysis and DNA was ethanol-extracted from pellets and quantified by PicoGreen dsDNA Quantification kit (Invitrogen, Melbourne, Australia). *In vitro* insulin secretion for each replicate was calculated as insulin concentration divided by DNA concentration. *In vitro* data for an animal was included in analyses provided that insulin secretion in incubations with KCl (test of maximal release) was greater than those obtained from incubations with epinephrine or at 0°C (inhibitory quality controls). Due to technical difficulties with some preparations, *in vitro* insulin secretion data was obtained successfully for 5 CON, 5 IUGR+Veh and 6 IUGR+Ex-4 lambs.

### Statistical analysis

Data for non-repeated measures on each animal were analysed by the mixed models procedure of SPSS for effects of treatment (fixed effect) and including dam as a random (block) effect in the model to account for common maternal environment in twins. Where treatment effects or trends were apparent (P<0.1), we then compared means by the LSD method, based on a priori questions to determine: 1. effects of IUGR (CON cf. IUGR+Veh groups), 2. effects of exendin-4 in IUGR lambs (IUGR+Veh cf. IUGR+Ex-4 groups), and 3. to assess whether exendin-4 restored values to those of controls (CON cf. IUGR+Ex-4 groups). We also confirmed these comparisons between IUGR+Veh and IUGR+Ex-4 groups using a paired t-test to compare twin siblings, and the significance of this test was consistent with that for LSD comparisons for all measures (data not shown). Neonatal growth patterns and glucose, insulin and insulin:glucose ratios overall and during 1^st^ phase (0–30 min) and 2^nd^ phase (30–210 min) of insulin secretion during the IVGTT were analysed by repeated measures for effects of treatment (between factor), time (within factor) and interactions, and including dam as a random (block) effect in the model to account for common maternal environment in twins. Glucose-stimulated in vitro insulin secretion was analysed by repeated measures for effects of treatment (between factor), glucose concentration (within factor) and interactions. Stimulation and inhibition of in vitro insulin secretion were analysed using repeated measures models for effects of treatment (between factor), stimulation (within factor, 11.1 mM glucose or KCl) or inhibition (within factor, 11.1 mM glucose or epinephrine) and interactions, and by mixed model as described above for incubations with individual secretagogues.

## Results

### Size at birth, neonatal growth and body composition

**Figure 1:**
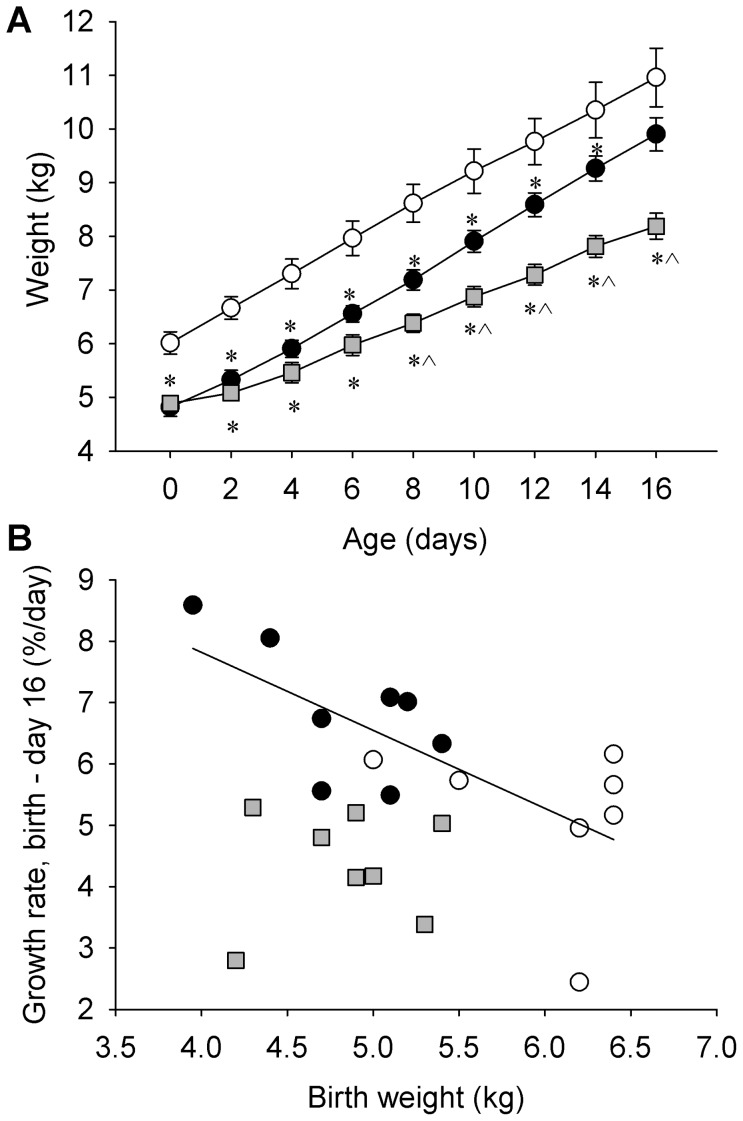
Effect of IUGR and neonatal exendin-4 treatment on neonatal growth. Neonatal exendin-4 treatment reduced weight of twin IUGR lambs from 8 days of age (A), and abolished the negative relationship between birth weight and neonatal fractional growth rate (B). CON (white circle) and IUGR+Veh (black circle) lambs were treated once daily with vehicle (0.5% methanol in saline s.c.) and IUGR+Ex-4 (gray square) lambs were treated once daily with exendin-4 (1 nmol.kg^−1^ s.c.). Data in Figure 1A are means ± SEM, and data in Figure 1B are individual animal outcomes. * different from CON (P<0.05), ? different from IUGR+Veh (P<0.05).

Lamb weight, abdominal circumference and body mass index at birth were reduced in twin lambs (all IUGR groups) compared to singleton lambs (each P<0.001, Table 1). Absolute and fractional growth rates for weight and abdominal circumference, and absolute but not fractional growth rate for shoulder height, differed with treatment (Table 1). IUGR+Veh lambs had higher FGR for weight and abdominal circumference than CON lambs (P = 0.022 and P = 0.001 respectively), and by d 16, there was no difference in weight between these two groups (Figure 1A). In control and IUGR+Veh lambs, FGR for weight increased as birth weight decreased (combined: R = −0.700, P = 0.002, n = 15; Figure 1B), whereas in IUGR+Ex4 lambs, neonatal FGR was not related to birth weight (P>0.3; Figure 1B). Neonatal exendin-4 treatment reduced neonatal growth rates (Table 1) including for weight (AGR_weight_, −35%, P<0.001), linear growth (AGR_shoulder height_, −20%, P = 0.031), and organ growth (AGR_abdominal circumference_, −30%, P = 0.007), and this group were lighter than CON and IUGR + Vehlambs at d16 (Figure 1). Neonatal exendin-4 reduced body weight (−18%, P = 0.016) and relative visceral fat mass (−57%, P<0.001) at post-mortem compared to IUGR+Veh lambs (Table 1). IUGR+Ex-4 lambs had lower absolute liver weights than CON (−28%, P = 0.001) or IUGR+Veh (−25%, P = 0.009) lambs, and lower relative liver weights (as a proportion of body weight) than IUGR+Veh lambs (−9%, P = 0.021). Absolute summed muscle mass was lower in IUGR+Veh lambs (−3.7%, P = 0.017) relative to CON, and was decreased by exendin-4 treatment relative to CON (−27%, P<0.001) and IUGR+Veh (−25%, P  = 0.004) groups. Relative summed muscle weight also tended to be lower in IUGR+Veh (−7.6%, P = 0.093) and was lower in IUGR+Ex-4 (−9.5%, P = 0.019) compared to CON lambs (Table 1).

**Table 1:** Effect of IUGR and neonatal exendin-4 on size at birth, postnatal growth and body composition in lambs.

	CON	IUGR+Veh	IUGR+Ex-4	Significance (treatment effect)
Number of animals	7	8	8	
Size at birth				
Birth weight (kg)	6.01±0.21	4.82±0.17*	4.84±0.15*	<0.001
Crown rump length (cm)	56.3±1.4	54.6±1.1	55.1±1.1	NS
Shoulder height (cm)	44.0±0.7	40.1±0.8*	40.9±0.7*	0.008
Abdominal circumference (cm)	40.1±0.4	35.1±0.9*	36.1±0.6*	<0.001
Body mass index (kg.m^−2^)	19.2±1.1	16.3±0.8*	16.0±0.7*	0.040
Neonatal Growth				
AGR_weight_ (g.day^−1^)	309±29	327±14	211±17*?	0.001
FGR_weight_ (%.day^−1^)	5.17±0.48	6.86±0.39	4.35±0.32?	0.001
AGR_shoulder height_ (cm.day^−1^)	0.390±0.027	0.507±0.037*	0.403±0.038?	0.030
FGR_shoulder height_ (%.day^−1^)	0.89±0.06	1.17±0.19	1.00±0.11	NS
AGR_abdominal circumference_ (cm.day^−1^)	0.473±0.075	0.782±0.042*	0.544±0.048?	0.002
FGR_abdominal circumference_ (%.day^−1^)	1.18±0.19	2.25±0.17*	1.52±0.15?	0.001
Postmortem (d 16)				
Body weight (kg)	11.0±0.5	10.1±0.3	8.33±0.25*?	<0.001
Total liver weight (g)	296±19	285±17	214±7*?	0.002
Total liver weight (% of body weight)	2.70±0.10	2.82±0.14	2.57±0.07?	NS
Summed muscle mass (g)	265±13	228±8*	183±9*?	<0.001
Summed muscle mass (% of body weight)	2.42±0.07	2.26±0.04	2.19±0.08*	0.055
Visceral fat (g)	132±19	118±11	41.7±6.3*?	<0.001
Visceral fat (% of body weight)	1.19±0.17	1.16±0.09	0.495±0.062*?	<0.001

Neonatal growth rates are from d 0 to 16. NS: P>0.1, * different from CON (P<0.05), ? different from IUGR+Veh (P<0.05).

### Insulin secretion, sensitivity and action

**Figure 2:**
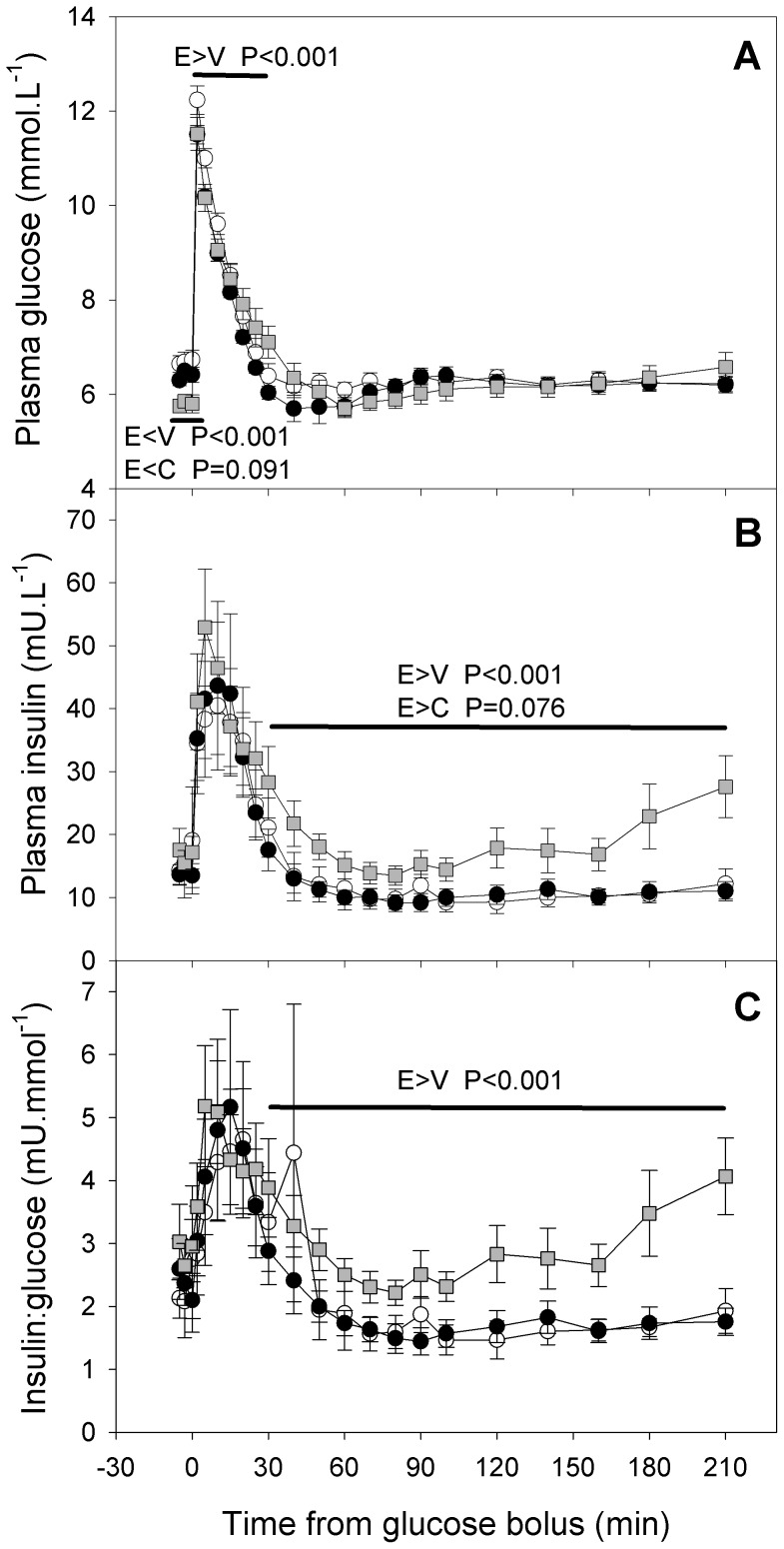
Effect of IUGR and neonatal exendin-4 treatment on in vivo metabolism in young lambs. Glucose tolerance (A), glucose-stimulated insulin secretion (B) and relative glucose-stimulated insulin secretion (C) were measured during an intravenous glucose tolerance test 14 days of age. CON (white circle, n = 7), IUGR+Veh (black circle, n = 8) and IUGR+Ex-4 (gray square, n = 8). Data are means ± SEM. * different from CON (P<0.05).

Fasting glucose and insulin levels, glucose tolerance and overall, 1^st^ phase and 2^nd^ phase insulin secretion in vivo were similar in IUGR+Veh and CON lambs (each P>0.1, Table 2). Fasting plasma glucose (d 14) was reduced in IUGR+Ex-4 lambs compared to CON (−10%, P = 0.022) and IUGR+Veh lambs (−9%, P = 0.019, Table 2). Conversely, glucose tolerance was impaired (increased glucose AUC) in IUGR+Ex-4 lambs overall (+132%, +156% respectively), during first phase insulin secretion (+41%, +57%), and during second phase insulin secretion compared to CON and IUGR+Veh lambs (each P≤0.02, Table 2). Across the whole of the IVGTT, and within the 1^st^ phase of insulin secretion, plasma glucose (Figure 2) changed with time (each P<0.001). Fasting plasma glucose in fasting samples was lower in IUGR+Ex4 than in IUGR+Veh lambs (P<0.001), and tended to be lower in IUGR+Ex4 than in CON lambs (P = 0.091). Conversely, plasma glucose during the 1^st^ phase of insulin secretion was higher in IUGR+Ex4 than in IUGR+Veh lambs (P<0.001), and plasma glucose during the 2^nd^ phase of insulin secretion did not differ between groups (P>0.3). The pattern of change in plasma glucose with time differed between groups overall (P<0.001) and during the 1^st^ phase of insulin secretion (P = 0.003). Fasting plasma insulin in absolute terms and relative to glucose, and insulin secretion (assessed relative to the glucose stimulus as AUC insulin/AUC glucose) did not differ between the groups (Table 2 and Figure 2). Plasma insulin (Figure 2) changed with time throughout the IVGTT (P<0.001), and within 1^st^ (P<0.001) and 2^nd^ phase (P = 0.008) of insulin response. The ratio of plasma insulin to glucose (Figure 2), an index of insulin secretion, similarly changed with time throughout the IVGTT (P<0.001), and within 1^st^ (P = 0.015) and 2^nd^ phase (P = 0.005) of insulin response. Plasma insulin concentrations and the ratio of plasma insulin to glucose ratios during the IVGTT (Figure 2) were higher in IUGR+Ex4 than in IUGR+Veh lambs overall (each P<0.001) and during the 2^nd^ phase of insulin secretion (each P<0.001), and did not differ between other treatment groups. IUGR+Ex-4 lambs had lower insulin sensitivity compared to CON (−44%, P = 0.004) and IUGR+Veh lambs (−46%, P = 0.002, Table 2). Basal and maximal insulin disposition indices did not differ between groups (Table 2).

**Table 2:** Effect of IUGR and neonatal exendin-4 on insulin action in lambs.

	CON	IUGR+Veh	IUGR+Ex-4	Significance (treatment effect)
Number of animals	7	8	8	
Fasting				
Plasma glucose (mmol.L^−1^)	6.47±0.26	6.40±0.11	5.81±0.12*?	0.008
Plasma insulin (mU.L^−1^)	20.4±6.0	15.4±2.2	16.4±2.2	NS
Plasma insulin:glucose (mU.mmol^−1^)	3.30±1.11	2.40±0.35	2.83±0.38	NS
AUC glucose (mmol.min.L^−1^)				
Total	62±6	56±3	143±28*?	0.003
1^st^ phase	60.9±5.4	54.8±2.9	86.2±5.7*?	<0.001
2^nd^ phase	1±1	1±1	57±24*?	0.017
AUC insulin (mU.min.L^−1^)				
Total	587±184	590±181	863±178	NS
1^st^ phase	499±133	579±180	650±128	NS
2^nd^ phase	88±61	12±6	213±119	NS
AUC insulin:AUC glucose (mU.mmol^−1^)				
Total	10.8±4.3	10.9±3.6	7.6±1.9	NS
1^st^ phase	8.9±2.8	10.7±3.5	7.8±1.6	NS
2^nd^ phase	26.5±25.9	0.3±0.3	9.6±2.8	NS
Insulin sensitivity (mg.L.mU^−1^.kg^−1^.min^−1^)	0.097±0.010	0.100±0.011	0.047±0.009*?	0.003
Basal IDI (mg.mL.kg^−2^.min^−2^)	69.7±31.2	39.5±5.5	28.4±10.0	NS
Maximal IDI (mg.mL.kg^−2^.min^−2^)	138±28	119±27	97±37	NS

Glucose and insulin AUC were measured during an IVGTT (0.25 g glucose.kg^−1^) at d 14. 1^st^ and 2^nd^ phase values for insulin and glucose were measured from 0–30 and from 30–210 minutes after glucose administration, respectively. Insulin sensitivity was measured during a hyperinsulinemic euglycemic clamp (2 mU insulin.kg^-1^.min^−1^) at d 12. NS: P>0.1, * different from CON (P<0.05), ? different from IUGR+Veh (P<0.05).

### Pancreas morphology and β-cell function

Absolute and relative pancreas weights, and numbers of β-cells per islet, β-cell volume density and absolute β-cell mass did not differ with treatment (Table 3). β-cell mass relative to body weight was greater in IUGR+Ex-4 lambs than CON lambs (+36%, P = 0.039, Table 3). IUGR+Ex-4 lambs also tended to have higher relative β-cell mass than IUGR+Veh lambs (+28%, P = 0.083, Table 3). Measures of β-cell function did not differ between treatments (Table 3).

**Table 3:** Effect of IUGR and neonatal exendin-4 on pancreas morphology and β-cell function.

	CON (7)	IUGR+Veh (8)	IUGR+Ex-4 (8)	Significance (treatment effect)
Pancreas morphology				
Pancreas weight (g)	10.8±1.5	8.53±0.93	7.90±0.55	NS
Pancreas (% of body weight)	0.103±0.019	0.085±0.009	0.096±0.007	NS
β-cell volume density	0.033±0.005	0.040±0.003	0.049±0.007	NS
β-cell mass (g)	0.326±0.038	0.345±0.054	0.387±0.055	NS
β-cell mass (% of body weight)	0.0030±0.0004	0.0034±0.0005	0.0047±0.0006*	0.070
Islet density (no.mm^−2^)	66.3±9.7	76.9±10.3	91.6±10.5	NS
β-cells/islets	10.9±1.4	10.5±1.3	12.9±1.3	NS
% of islets with <5β-cells	27.7±6.3	23.8±6.4	31.3±6.7	NS
β-cell function				
Insulin secretion (AUC ins) per β-cell mass (mU.min.L^−1^.g^−1^)	1682±413	1944±588	2190±286	NS
Basal IDI per β-cell mass (mg.mL.kg^−2^.min^−2^.g^−1^)	187±60	129±25	85.4±26.5	NS
Max IDI per β-cell mass(mg.mL.kg^−2^.min^−2^.g^−1^)	441±85	389±89	269±94	NS

NS: P>0.1, * different from CON (P<0.05).

### *In vitro* β-cell secretory function

**Figure 3:**
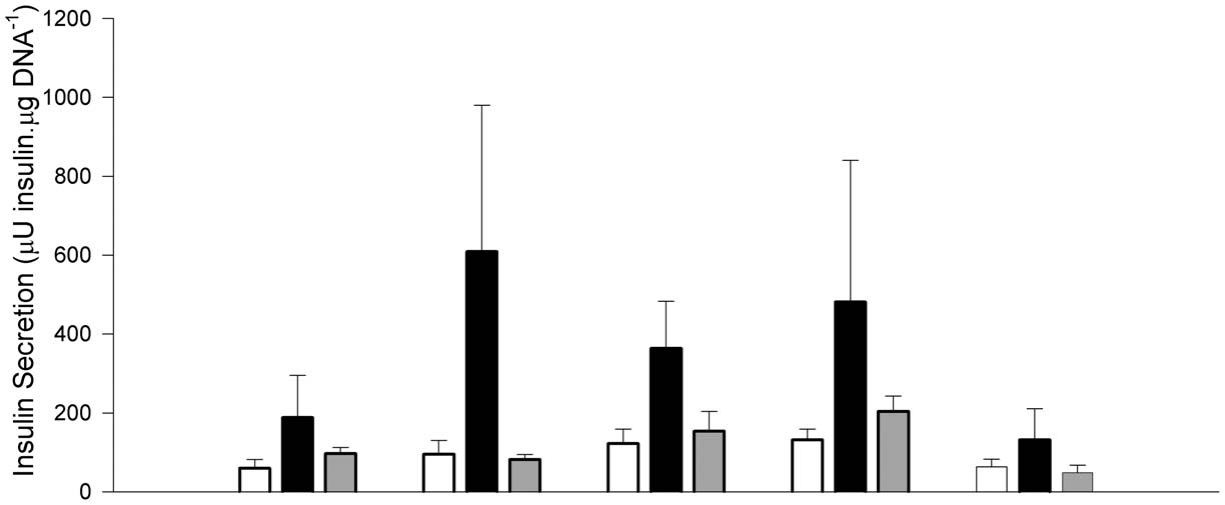
Effect of IUGR and neonatal exendin-4 treatment on in vitro insulin secretion from isolated islets in response to glucose and potassium chloride. CON (white bar, n = 5), IUGR+Veh (black bar, n = 5) and IUGR+Ex-4 (gray bar, n = 6). Data are means ± SEM. Specific contrasts: * P<0.05, ^#^ P<0.10.

Islet insulin secretion (Figure 3) increased with increasing glucose concentration between 0 and 11.1 mM overall (P = 0.006). Glucose-stimulated insulin secretion tended to be higher overall in IUGR+Veh compared to CON lambs (+420%, P = 0.081), did not differ between IUGR+Ex4 lambs and CON lambs (P = 0.9) and tended to be higher in IUGR+Veh lambs than in IUGR+Ex4 lambs (+20%, P = 0.087). At the highest glucose concentration (11.1 mM), IUGR +Vehlambs had higher insulin secretion than CON lambs (+66%, P = 0.046) and tended to have higher insulin secretion than IUGR+Ex-4 lambs (+58%, P = 0.066 respectively, Figure 3). Within each group of lambs, in vitro insulin secretion at 11.1 mM glucose was between 1.6 and 2-fold higher than that at 0 mM glucose (Figure 3). In vitro insulin secretion was similar from islets incubated with 15 mM KCl or 11.1 mM glucose (P>0.5), and the response to KCl was similar between treatments (P>0.8). In vitro insulin secretion was suppressed by epinephrine treatment compared to glucose-stimulated insulin secretion (−62%, P = 0.001). Suppression of glucose-stimulated insulin secretion by epinephrine was greater in IUGR+Veh than CON lambs in absolute terms (−173 cf. −47 µU insulin/µgDNA, P = 0.044), but not as a proportion of insulin secretion in the absence of epinephrine (−28.8% cf. −7.8%, P = 0.274). Epinephrine suppression of glucose-stimulated insulin secretion was similar in islets from IUGR-Ex4 to that in other groups (P>0.1 for each). Lysine-, arginine- and leucine-stimulated in vitro islet insulin secretion did not differ between treatment groups (each P>0.3, data not shown).

## Discussion

In the present study, twin IUGR lambs caught up in weight by 16 d of age, and had normal *in vivo* insulin action in their second week of life, with similar β-cell mass to singleton control lambs. Glucose-stimulated *in vitro* insulin secretion was increased in the IUGR twin lamb relative to controls, suggesting up-regulated β-cell function at this age. Daily exendin-4 treatment of twin IUGR lambs during neonatal life prevented catch-up growth and fat accumulation, and normalised *in vitro* insulin secretion from their islets, relative to untreated IUGR twins, which may retain adaptive capacity for later life. Glucose tolerance of IUGR lambs was impaired during exendin-4 treatment however, reflecting decreased insulin sensitivity and occurred despite greater *in vivo* insulin secretion. This may be due to central actions of exendin-4 to inhibit food intake and insulin sensitivity [37], [38], [39]. Nevertheless, the reduction in fat accumulation and normalised β-cell action *in vitro* of IUGR lambs suggest that neonatal exendin-4 might have beneficial effects on insulin-regulated glucose homeostasis in later life. These outcomes also demonstrate the biological activity of exendin-4 for the first time in the sheep, at least in the context of individuals who had undergone growth-restriction before birth.

We found similar growth and metabolic responses to IUGR induced by twinning in this study to those seen previously after IUGR induced by restriction of placental growth and function (PR) in sheep. Like the PR lamb, the twin IUGR lambs in the present study experienced accelerated neonatal catch-up growth, achieving a normal body weight by 16 d of age in this study and by 30 d of age in our studies in PR lambs [40]. Accelerated fat deposition occurs during accelerated neonatal growth, and in humans catch-up growth is a risk factor for later obesity [41]. PR lambs have fat stores proportionate to their reduced body weight in late gestation [42], and similar to our twin lambs at 16 days in the present study, fat mass relative to body weight is similar in PR and CON lambs at 21 days despite their catch up growth [43]. By 43 days of age, however, the accelerated fat deposition results in greater visceral fat in PR lambs than their control counterparts [40]. Small size at birth in humans consistently induces insulin resistance in adults and adolescents [44], but this is preceded by enhanced insulin sensitivity in neonates, which reverses to resistance in association with catch-up growth in the first few years of life [45]. There is similar evidence of a reversal from insulin sensitivity to insulin resistance in the lamb following IUGR induced by restriction of placental growth and function (PR). The young PR lamb at 21 days of age has increased expression of insulin receptors and insulin signalling molecules in skeletal muscle [46], although *in vivo* insulin action was not measured. At 30 days, glucose tolerance of PR lambs is normal, despite decreased insulin action caused by falls in both *in vivo* insulin secretion and insulin sensitivity [40], [47]. The latter reflects decreased expression of insulin-signalling pathways in skeletal muscle [47]. Impaired glucose tolerance and elevated fasting glucose emerge by 1 year of age in IUGR sheep [48]. The normal insulin sensitivity and glucose tolerance seen here in the twin IUGR lamb may therefore reflect the beginnings of the reversal from insulin sensitivity to insulin resistance occurring during the neonatal catch-up growth they are experiencing at this age.

Neither IUGR nor neonatal exendin-4 treatment in IUGR lambs altered relative β-cell mass at 16 days in the present study, consistent with the lack of effect of PR and neonatal exendin-4 treatment on β-cell mass in young postnatal rats at 2 weeks of age [49]. In the rat, reduced β-cell mass after IUGR emerges by 3 months of age in young adults, and neonatal exendin-4 treatment normalises adult β-cell mass at this age in this model [49]. We hypothesise that these beneficial effects of exendin-4 treatment after IUGR might also emerge with ageing in the sheep. This lack of an immediate response may also reflect the collection of pancreas soon after completion of exendin-4 treatment here and in PR rats. Previous rodent studies have reported increased β-cell replication after similar exendin-4 treatment durations, but differences in β-cell mass are sometimes not apparent until several weeks later [50], [51]. Many of the actions of exendin-4 and GLP-1 on insulin secretion are mediated via stimulation of *Pdx-1* expression, a transcription factor important for regulation of β-cell mass as well as function, and which is required for plasticity of β-cell mass and function to increase insulin secretion in response to demand. In the PR rat, prevention of later diabetes following neonatal exendin-4 treatment reflects reversal of epigenetic changes induced by PR in the *Pdx-1* promoter by late gestation, that normally worsen with age and lead to decreased *Pdx-1* expression, loss of β-cell function and subsequent loss of β-cell mass postnatally [15], [16], [19]. Intriguingly, although neonatal exendin-4 induces epigenetic changes such as increased acetylation and lysine 4 trimethylation at histone H3 in control as well as PR rat juveniles, it only increases *Pdx-1* expression and β-cell mass and improves glucose tolerance in the PR progeny [15], [16], [19]. Indeed, the *Pdx-*1 promotor becomes methylated and hence partially silenced by adulthood in untreated PR rat progeny, but not in control progeny regardless of exendin-4 treatment, which implies that the levels of histone 3 acetylation and lysine 4 trimethylation in untreated control progeny is already sufficient to prevent later promoter methylation [16]. We do not yet know whether neonatal exendin-4 treatment will affect outcomes in control sheep progeny, as the aim of the present study was to evaluate its efficacy only in the context of IUGR. Whether neonatal exendin-4 acts similarly in the IUGR lamb as in the PR rat, by reversing epigenetic changes in the *Pdx-1* promotor and improves adult β-cell mass and function to delay or prevent the subsequent loss of insulin secretory capacity observed after IUGR in young adult male sheep [11] remains to be determined, and will require separate animal cohorts with long-term follow-up of functional and molecular outcomes

In this study, the decrease in fasting plasma glucose (∼9%) and more sustained insulin secretion during exendin-4 treatment in the IUGR neonatal lamb compared to untreated IUGR siblings were generally consistent with responses to exendin-4 in rodents and humans. Medium- to long-term exendin-4 treatment in human T2DM patients (daily 5–10 µg injections for 30 and 82 weeks) [52], [53], [54], in the obese diabetic *db/db* mouse (1 nmol.kg^−1^.d^−1^ as daily injections for 14 days) [55], and in the obese *ob/ob* mouse (20 µg.kg^−1^.d^−1^, ∼ 5 nmol.kg^−1^.d^−1^ as twice daily injections for 60 days) [56] reduces fasting blood glucose as well as HbA1c, a marker of chronic hyperglycemia. Earlier studies in humans also demonstrated acute decreases in fasting and post-prandial glucose concentrations after a single exendin-4 dose and after 5 days of twice daily injections with 5 µg exendin-4 [57]. Infusions with GLP-1 and chronic exendin-4 treatment enhance post-prandial and glucose-stimulated insulin secretion in human patients with T2DM, including restoration of 1^st^ phase insulin secretion response to glucose, and sustained elevation of 2^nd^ phase insulin secretion in T2DM patients [57], [58], [59]. In the diabetic rat, four weeks of twice-daily exendin-4 injections (105 pmol.kg^−1^) increased 1^st^ and 2^nd^ phase insulin secretion during a hyperglycemic clamp [60]. Whilst we similarly observed increases in second phase insulin secretion in IUGR lambs during exendin-4 treatment, their first phase insulin secretion was unchanged. This apparent difference may be because first phase insulin secretion is normal in the IUGR lamb at this age, whereas previous reports of increased first phase insulin responses after exendin-4 or GLP-1 treatment have all been in the context of diabetes, when first phase secretion is impaired. The effects of exendin-4 on insulin secretion in IUGR sheep AFTER cessation of treatment remain to be investigated.

We performed *in vitro* testing to measure intrinsic β-cell function independent of systemic input from endocrine and nervous systems [36]. In this study, IUGR neonatal lambs had enhanced *in vitro* glucose-stimulated insulin secretion, or β-cell hypersecretion relative to control lambs, which occurs in obese individuals, as well as early in the pathogenesis of type 2 diabetes [61], [62], [63]. Interestingly, exendin-4 treatment of IUGR lambs abolished this *in vitro* insulin hypersecretion from isolated islets, suggesting some normalisation of intrinsic β-cell function and its determinants. Together with increased β-cell mass, this suggests that neonatal exendin-4 may improve insulin secretory capacity after IUGR.

In contrast with the improved insulin sensitivity seen after chronic GLP-1 or exendin-4 treatment in human patients with extreme obesity [64] or T2DM [65], insulin sensitivity was profoundly decreased on the 11^th^ day of exendin-4 treatment in neonatal IUGR lambs, relative to their untreated IUGR littermates. In studies of exendin-4 action in rodents, direct measures and calculated indices of insulin sensitivity have either been increased [56], [60], [66], or not altered [55], immediately following or during chronic (2 – 9 weeks) exendin-4 treatment. We propose that the differential effects of exendin-4 on insulin sensitivity may depend on whether the latter is assessed during treatment or after, whether the subjects are obese and on their developmental stage and growth rate. Exendin-4 reduced weight gain in the IUGR lambs in the present study as well as in PR rat neonates [15], consistent with its actions including decreased food and caloric intake, reduced gastric emptying and induced weight loss or slowed weight gain in mice and rats [67], [68] and in adolescent and adult humans [52], [53], [54], [64], [69], [70], [71]. It appears that restricted nutrition reduces insulin sensitivity in growing animals, possibly partly due to reduced mass of insulin-responsive tissues, whereas in older or obese animals the net effect of restricted feeding and consequently reduced fatness is to increase insulin sensitivity. Thus, feed restriction *increases* insulin-stimulated glucose metabolism and insulin sensitivity in adult sheep [72], but decreases insulin-stimulated glucose uptake in muscle of young growing pigs [73]. In mice, exendin-4 can cross the blood-brain barrier [74], and acts centrally to suppress femoral blood flow and whole body insulin sensitivity, via the GLP-1 receptor and activation of PKC-δ signalling pathways in the hypothalamus [37], [38], suggesting an additional mechanism for decreased peripheral insulin sensitivity during exendin-4 treatment. As a consequence of their reduced insulin sensitivity, and despite the increased 2^nd^ phase insulin secretion that maintained insulin disposition, glucose tolerance was impaired in IUGR+Ex-4 lambs compared to IUGR+Veh and CON lambs. This contrasts with improved glucose tolerance observed 24 h after completion of medium- to long-term exendin-4 treatment in mature rats [56], [59], [60], [66], during continued long-term exendin-4 treatment in β-cell depleted rats [75], and acutely in T2DM human patients [76]. In some of these studies, the improved glucose tolerance during or after exendin-4 treatment reflects marked improvement of deficient insulin secretion due to stimulation of β-cell regeneration [75] or up-regulation of β-cell function in T2DM patients [76]. Long-term exendin-4 treatment increases insulin sensitivity in obese humans, genetically-obese rodents and diabetic humans and rodents, measured either during or 16–24 h after completion of treatment [56], [64], [77], [78]. Improved whole-body insulin sensitivity is probably also due to improvements in hepatic insulin sensitivity, with lower post-prandial endogenous glucose production after or during exendin-4 treatment [79]. To our knowledge, this is the first study of the effects of exendin-4 on insulin action treatment in young growing animals. Further studies are needed to define the underlying mechanisms for their reduced insulin sensitivity during treatment.

The profound reduction in visceral fat deposition after IUGR in response to exendin-4 is also of particular potential importance for later glucose homeostasis, given that obesity and particularly visceral fat deposition are strong risk factors for impaired glucose tolerance and T2DM [80], [81]. In the PR rat, neonatal exendin-4 reduces weight gain in conjunction with prevention of later diabetes, and this may particularly reduce the risk of T2DM in IUGR subjects [15], since catch-up growth after IUGR is a risk factor for T2DM and for adult obesity [82], [83]. Intriguingly, neonatal exendin-4 treatment abolished the negative relationship between birth weight and fractional growth rate in IUGR lambs in the current study. In contrast to its metabolic effects, exendin-4 reduced neonatal growth and adult size in both control and PR rat progeny [15]. This suggests that exendin-4 may act in part, but not only, via the pathway/s responsible for catch-up after IUGR, which include neonatal hyperphagia, elevated insulin sensitivity and increased abundance of thyroid hormones in IUGR lambs [40], [84], [85]. Longer-term evaluations of growth and composition after cessation of exendin-4 are needed to determine whether this decrease in central adiposity persists in the IUGR sheep.

In conclusion, neonatal exendin-4 treatment increased 2^nd^ phase insulin secretion *in vivo*, normalised *in vitro* insulin secretion and decreased visceral fat at the end of treatment in the IUGR lamb. Neonatal exendin-4 treatment also improves insulin secretion and glucose tolerance in adolescent and adult rat progeny following IUGR, preventing development of diabetes in these animals [15], although the effects during treatment were not measured in the latter study. Investigation of the long-term effects of neonatal exendin-4 on glucose homeostasis and insulin action in the IUGR lamb into adulthood should be a priority for the future.

## References

[1] Newsome CA, Shiell AW, Fall CHD, Phillips DIW, Shier R, et al (2003) Is birth weight related to later glucose and insulin metabolism?-a systematic review. Diab Med 20: 339–348. 10.1046/j.1464-5491.2003.00871.x 12752481

[2] Whincup PH, Kaye SJ, Owen CG, Huxley R, Cook DG, et al (2008) Birth weight and risk of type 2 diabetes: A systematic review. JAMA 300: 2886–2897. 19109117 10.1001/jama.2008.886

[3] Kaijser M, Edstedt Bonamy A-K, Akre O, Cnattingius S, Granath F, et al (2009) Perinatal risk factors for diabetes in later life. Diabetes 58: 523–526. 19066311 10.2337/db08-0558 PMC2646049

[4] Eriksson M, Wallander MA, Krakau I, Wedel H, Svardsudd K (2004) Birth weight and cardiovascular risk factors in a cohort followed until 80 years of age: the study of men born in 1913. J Internal Med 255: 236–246. 14746561 10.1046/j.1365-2796.2003.01289.x

[5] Jensen CB, Storgaard H, Dela F, Holst JJ, Madsbad S, et al (2002) Early differential defects of insulin secretion and action in 19-year-old Caucasian men who had low birth weight. Diabetes 51: 1271–1280. 11916955 10.2337/diabetes.51.4.1271

[6] Veening MA, van Weissenbruch MM, Heine RJ, Delemarre-van de Waal HA (2003) β-cell capacity and insulin sensitivity in prepubertal children born small for gestational age: Influence of body size during childhood. Diabetes 52: 1756–1760. 12829643 10.2337/diabetes.52.7.1756

[7] Mericq V, Ong KK, Bazaes R, Peña V, Avila A, et al (2005) Longitudinal changes in insulin sensitivity and secretion from birth to age three years in small- and appropriate-for-gestational-age children. Diabetologia 48: 2609–2614. 16283238 10.1007/s00125-005-0036-z

[8] Robinson JS, Owens JA (1996) Pathophysiology of intrauterine growth failure. In: Gluckman PD, Heymann MA, editors. Pediatrics and Perinatology The Scientific Basis. 2 ed. London Arnold.pp. 290–297.

[9] De Blasio MJ, Gatford KL, McMillen IC, Robinson JS, Owens JA (2007) Placental restriction of fetal growth increases insulin action, growth, and adiposity in the young lamb. Endocrinology 148: 1350–1358. 17110432 10.1210/en.2006-0653

[10] De Blasio MJ, Gatford KL, Robinson JS, Owens JA (2007) Placental restriction of fetal growth reduces size at birth and alters postnatal growth, feeding activity, and adiposity in the young lamb. Am J Physiol Regul Integr Comp Physiol 292: R875–886. 17023666 10.1152/ajpregu.00430.2006

[11] Owens JA, Thavaneswaran P, De Blasio MJ, McMillen IC, Robinson JS, et al (2007) Sex-specific effects of placental restriction on components of the metabolic syndrome in young adult sheep. Am J Physiol Endocrinol Metab 292: E1879–1889. 17327366 10.1152/ajpendo.00706.2006

[12] Gatford KL, Mohammad SNB, Harland ML, De Blasio MJ, Fowden AL, et al (2008) Impaired β-cell function and inadequate compensatory increases in β-cell mass after intrauterine growth restriction in sheep. Endocrinology 149: 5118–5127. 18535100 10.1210/en.2008-0233

[13] Simmons RA, Templeton LJ, Gertz SJ (2001) Intrauterine growth retardation leads to the development of type 2 diabetes in the rat. Diabetes 50: 2279–2286. 11574409 10.2337/diabetes.50.10.2279

[14] Simmons RA, Suponitsky-Kroyter I, Selak MA (2005) Progressive accumulation of mitochondrial DNA mutations and decline in mitochondrial function lead to β-cell failure. J Biol Chem 280: 28785–28791. 15946949 10.1074/jbc.M505695200

[15] Stoffers DA, Desai BM, DeLeon DD, Simmons RA (2003) Neonatal exendin-4 prevents the development of diabetes in the intrauterine growth retarded rat. Diabetes 52: 734–740. 12606515 10.2337/diabetes.52.3.734

[16] Pinney S, Jaeckle Santos L, Han Y, Stoffers D, Simmons R (2011) Exendin-4 increases histone acetylase activity and reverses epigenetic modifications that silence *Pdx-1* in the intrauterine growth retarded rat. Diabetologia 54: 2606–2614. 21779870 10.1007/s00125-011-2250-1 PMC4461231

[17] Brissova M, Blaha M, Spear C, Nicholson W, Radhika A, et al (2005) Reduced PDX-1 expression impairs islet response to insulin resistance and worsens glucose homeostasis. Am J Physiol Endocrinol Metab 288: E707–714. 15562255 10.1152/ajpendo.00252.2004

[18] Kulkarni RN, Jhala US, Winnay JN, Krajewski S, Montminy M, et al (2004) PDX-1 haploinsufficiency limits the compensatory islet hyperplasia that occurs in response to insulin resistance. J Clin Invest 114: E828–836. 10.1172/JCI21845 PMC516265 15372107

[19] Park JH, Stoffers DA, Nicholls RD, Simmons RA (2008) Development of type 2 diabetes following intrauterine growth retardation in rats is associated with progressive epigenetic silencing of *Pdx1* . J Clin Invest 118: E2316–2324. 10.1172/JCI33655 PMC2373422 18464933

[20] Reddy S, Elliott RB (1988) Ontogenic development of peptide hormones in the mammalian fetal pancreas. Experientia 44: E1–9. 10.1007/BF01960221 2895013

[21] Kassem SA, Ariel I, Thornton PS, Scheimberg I, Glaser B (2000) β-cell proliferation and apoptosis in the developing normal human pancreas and in hyperinsulinism of infancy. Diabetes 49: 1325–1333. 10923633 10.2337/diabetes.49.8.1325

[22] Piper K, Brickwood S, Turnpenny LW, Cameron IT, Ball SG, et al (2004) Beta cell differentiation during early human pancreas development. J Endocrinol 181: 11–23. 15072563 10.1677/joe.0.1810011

[23] Limesand SW, Jensen J, Hutton JC, Hay WW Jr (2005) Diminished β-cell replication contributes to reduced β-cell mass in fetal sheep with intrauterine growth restriction. Am J Physiol Regul Integr Comp Physiol 288: R1297–1305. 15650129 10.1152/ajpregu.00494.2004

[24] Otonkoski T, Andersson S, Knip M, O S (1988) Maturation of insulin response to glucose during human fetal and neonatal development. Studies with perifusion of pancreatic isletlike cell clusters. Diabetes 37: 286–291. 3286329 10.2337/diab.37.3.286

[25] Bassett JM (1977) Glucagon, insulin and glucose homeostasis in the fetal lamb. Ann Rech Vet 8: 362–373. 615510

[26] Fowden AL (1980) Effects of arginine and glucose on the release of insulin in the sheep fetus. J Endocrinol 87: E293–301. 10.1677/joe.0.0850121 6993600

[27] Rozance PJ, Limesand SW, Hay WW (2006) Decreased nutrient-stimulated insulin secretion in chronically hypoglycemic late-gestation fetal sheep is due to an intrinsic islet defect. Am J Physiol Endocrinol Metab 291: E404–E411. 16569758 10.1152/ajpendo.00643.2005

[28] Scaglia L, Cahill CJ, Finegood DT, Bonner-Weir S (1997) Apoptosis participates in the remodeling of the endocrine pancreas in the neonatal rat. Endocrinology 138: 1736–1741. 9075738 10.1210/endo.138.4.5069

[29] Petrik J, Arany E, McDonald TJ, Hill DJ (1998) Apoptosis in the pancreatic islet cells of the neonatal rat is associated with a reduced expression of insulin-like growth factor II that may act as a survival factor. Endocrinology 139: 2994–3004. 9607811 10.1210/endo.139.6.6042

[30] Petrik J, Reusens B, Arany E, Remacle C, Coelho C, et al (1999) A low protein diet alters the balance of islet cell replication and apoptosis in the fetal and neonatal rat and is associated with a reduced pancreatic expression of insulin-like growth factor-II. Endocrinology 140: 4861–4873. 10499546 10.1210/endo.140.10.7042

[31] Navarro-Tableros V, Fiordelisio T, Hernández-Cruz A, Hiriart M (2007) Physiological development of insulin secretion, calcium channels, and GLUT2 expression of pancreatic rat β-cells. Am J Physiol Endocrinol Metab 292: E1018–E1029. 17148757 10.1152/ajpendo.00457.2006

[32] Tan C, Tuch BE, Tu J, Brown SA (2002) Role of NADH shuttles in glucose-induced insulin secretion from fetal β-cells. Diabetes 51: 2989–2996. 12351438 10.2337/diabetes.51.10.2989

[33] National Health and Medical Research Council of Australia (2004) Australian code of practice for the care and use of animals for scientific purposes, 7th edition. Canberra: Australian Government Publishing Service. 82 p.

[34] Thompson GE (1983) The intake of milk by suckled, newborn lambs and the effects of twinning and cold exposure. British J Nutr 50: 151–156. 10.1079/bjn19830082 6683972

[35] Gatford KL, De Blasio MJ, Thavaneswaran P, Robinson JS, McMillen IC, et al (2004) Postnatal ontogeny of glucose homeostasis and insulin action in sheep. Am J Physiol Endocrinol Metab 286: E1050–1059. 14761875 10.1152/ajpendo.00340.2003

[36] Limesand SW, Rozance PJ, Zerbe GO, Hutton JC, Hay WW Jr (2006) Attenuated insulin release and storage in fetal sheep pancreatic islets with intrauterine growth restriction. Endocrinology 147: 1488–1497. 16339204 10.1210/en.2005-0900

[37] Cabou C, Campistron G, Marsollier N, Leloup C, Cruciani-Guglielmacci C, et al (2008) Brain glucagon-like peptide-1 regulates arterial blood flow, heart rate, and insulin sensitivity. Diabetes 57: 2577–2587. 18633100 10.2337/db08-0121 PMC2551665

[38] Cabou C, Vachoux C, Campistron G, Drucker DJ, Burcelin R (2011) Brain GLP-1 signaling regulates femoral artery blood flow and insulin sensitivity through hypothalamic PKC-δ. Diabetes 60: 2245–2256. 21810595 10.2337/db11-0464 PMC3161335

[39] Imeryüz N, Yeğen BÇ, Bozkurt A, Coşkun T, Villanueva-Peñacarrillo ML, et al (1997) Glucagon-like peptide-1 inhibits gastric emptying via vagal afferent-mediated central mechanisms. American Journal of Physiology - Gastrointestinal and Liver Physiology 273: G920–G927. 10.1152/ajpgi.1997.273.4.G920 9357836

[40] De Blasio MJ, Gatford KL, McMillen IC, Robinson JS, Owens JA (2007) Placental restriction of fetal growth increases insulin action, growth and adiposity in the young lamb. Endocrinology 148: 1350–1358. 17110432 10.1210/en.2006-0653

[41] Ong KKL, Ahmed ML, Emmett PM, Preece MA, Dunger DB (2000) Association between postnatal catch-up growth and obesity in childhood: prospective cohort study. British Medical Journal 320: 967–971. 10753147 10.1136/bmj.320.7240.967 PMC27335

[42] Duffield JA, Vuocolo T, Tellam R, Yuen BS, Muhlhausler BS, et al (2008) Placental restriction of fetal growth decreases IGF1 and leptin mRNA expression in the perirenal adipose tissue of late gestation fetal sheep. American Journal of Physiology 294: R1413–R1419. 18272661 10.1152/ajpregu.00787.2007

[43] Duffield JA, Vuocolo T, Tellam R, McFarlane JR, Kauter KG, et al (2009) Intrauterine growth restriction and the sex specific programming of leptin and peroxisome proliferator-activated receptor gamma (PPARgamma) mRNA expression in visceral fat in the lamb. Pediatric Research 66: 59–65. 19342985 10.1203/PDR.0b013e3181a7c121

[44] Newsome CA, Shiell AW, Fall CHD, Phillips DIW, Shier R, et al (2003) Is birth weight related to later glucose and insulin metabolism? - a systemic review. Diabetic Medicine 20: 339–348. 12752481 10.1046/j.1464-5491.2003.00871.x

[45] Mericq V, Ong KK, Bazaes R, Pena V, Avila A, et al (2005) Longitudinal changes in insulin sensitivity and secretion from birth to age three years in small- and appropriate-for-gestational-age children. Diabetologia 48: 2609–2614. 16283238 10.1007/s00125-005-0036-z

[46] Muhlhausler BS, Duffield JA, Ozanne SE, Pilgrim C, Turner N, et al (2009) The transition from fetal growth restriction to accelerated postnatal growth: a potential role for insulin signalling in skeletal muscle. The Journal of Physiology 587: 4199–4211. 19622603 10.1113/jphysiol.2009.173161 PMC2754360

[47] De Blasio MJ, Gatford KL, Harland ML, Robinson JS, Owens JA (2012) Placental restriction reduces insulin sensitivity and expression of insulin signaling and glucose transporter genes in skeletal muscle, but not liver, in young sheep. Endocrinology 153: 2142–2151. 22434080 10.1210/en.2011-1955

[48] Owens JA, Harland ML, De Blasio MJ, Gatford KL, Robinson JS (2007) Restriction of placental and fetal growth reduces expression of insulin signalling and glucose transporter genes in skeletal muscle of young lambs. Early Human Development 83: S134 (abstract)..

[49] Stoffers DA, Desai BM, De Leon DD, Simmons RA (2003) Neonatal exendin-4 prevents the development of diabetes in the intrauterine growth retarded rat. Diabetes 52: 734–740. 12606515 10.2337/diabetes.52.3.734

[50] Doyle ME, Egan JM (2001) Glucagon-like peptide-1. Recent Prog Horm Res 56: 377–400. 11237222 10.1210/rp.56.1.377

[51] Garber AJ (2011) Incretin effects on β-cell function, replication, and mass: the human perspective. Diabetes Care pp. S258+. 10.2337/dc11-s230 PMC3632189 21525465

[52] Blonde L, Klein EJ, Han J, Zhang B, Mac SM, et al (2006) Interim analysis of the effects of exenatide treatment on A1C, weight and cardiovascular risk factors over 82 weeks in 314 overweight patients with type 2 diabetes. Diabetes Obesity Metab 8: 436–447. 10.1111/j.1463-1326.2006.00602.x 16776751

[53] Buse JB, Henry RR, Han J, Kim DD, Fineman MS, et al (2004) Effects of exenatide (Exendin-4) on glycemic control over 30 weeks in sulfonylurea-treated patients with type 2 diabetes. Diabetes Care 27: 2628–2635. 15504997 10.2337/diacare.27.11.2628

[54] DeFronzo RA, Ratner RE, Han J, Kim DD, Fineman MS, et al (2005) Effects of exenatide (exendin-4) on glycemic control and weight over 30 weeks in metformin-treated patients with type 2 diabetes. Diabetes Care 28: 1092–1100. 15855572 10.2337/diacare.28.5.1092

[55] Wang QW, Brubaker PLB (2002) Glucagon-like peptide-1 treatment delays the onset of diabetes in 8 week-old *db/db* mice. Diabetologia 45: 1263–1273. 12242459 10.1007/s00125-002-0828-3

[56] Ding X, Saxena NK, Lin S, Gupta N, Anania FA (2006) Exendin-4, a glucagon-like protein-1 (GLP-1) receptor agonist, reverses hepatic steatosis in *ob*/*ob* mice. Hepatology 43: 173–181. 16374859 10.1002/hep.21006 PMC2925424

[57] Kolterman OG, Buse JB, Fineman MS, Gaines E, Heintz S, et al (2003) Synthetic exendin-4 (exenatide) significantly reduces postprandial and fasting plasma glucose in subjects with type 2 diabetes. J Clin Endocrinol Metab 88: 3082–3089. 12843147 10.1210/jc.2002-021545

[58] Nauck MA, Heimesaat MM, Orskov C, Holst JJ, Ebert R, et al (1993) Preserved incretin activity of glucagon-like peptide 1 [7-36 amide] but not of synthetic human gastric inhibitory polypeptide in patients with type-2 diabetes mellitus. J Clin Invest 91: 301–307. 8423228 10.1172/JCI116186 PMC330027

[59] Fehse F, Trautmann M, Holst JJ, Halseth AE, Nanayakkara N, et al (2005) Exenatide augments first- and second-phase insulin secretion in response to intravenous glucose in subjects with type 2 diabetes. J Clin Endocrinol Metab 90: 5991–5997. 16144950 10.1210/jc.2005-1093

[60] Kwon DY, Kim YS, Ahn IS, Kim DS, Kang S, et al (2009) Exendin-4 potentiates insulinotropic action partly via increasing β-cell proliferation and neogenesis and decreasing apoptosis in association with the attenuation of endoplasmic reticulum stress in islets of diabetic rats. J Pharmacol Sci 111: 361–371. 20019445 10.1254/jphs.09178fp

[61] Jahr H, Ratzmann KP, Beckert R, Besch W, Hahn HJ (1983) Enhanced synthesis, storage, and secretion of insulin in pancreatic islets derived from obese subjects. Metabolism 32: 1101–1106. 6358778 10.1016/0026-0495(83)90055-0

[62] Hansen BC, Bodkin NL (1990) Beta-cell hyperresponsiveness: earliest event in development of diabetes in monkeys. Am J Physiol Regul Integr Comp Physiol 259: R612–R617. 10.1152/ajpregu.1990.259.3.R612 2204282

[63] Jetton TL, Lausier J, LaRock K, Trotman WE, Larmie B, et al (2005) Mechanisms of compensatory β-cell growth in insulin-resistant rats. Diabetes 54: 2294–2304. 16046294 10.2337/diabetes.54.8.2294

[64] Kelly AS, Metzig AM, Rudser KD, Fitch AK, Fox CK, et al (2012) Exenatide as a weight-loss therapy in extreme pediatric obesity: A randomized, controlled pilot study. Obesity 20: 364–370. 22076596 10.1038/oby.2011.337 PMC3684414

[65] Zander M, Madsbad S, Madsen JL, Holst JJ (2002) Effect of 6-week course of glucagon-like peptide 1 on glycaemic control, insulin sensitivity, and β-cell function in type 2 diabetes: a parallel-group study. Lancet 359: 824–830. 11897280 10.1016/S0140-6736(02)07952-7

[66] Gedulin BR, Nikoulina SE, Smith PA, Gedulin G, Nielsen LL, et al (2005) Exenatide (exendin-4) improves insulin sensitivity and β-cell mass in insulin-resistant obese *fa/fa* Zucker rats independent of glycemia and body weight. Endocrinology 146: 2069–2076. 15618356 10.1210/en.2004-1349

[67] Li L, Yang G, Li Q, Tan X, Liu H, et al (2008) Exenatide prevents fat-induced insulin resistance and raises adiponectin expression and plasma levels. Diabetes Obesity Metab 10: 921–930. 10.1111/j.1463-1326.2007.00832.x 18093209

[68] Arakawa M, Ebato C, Mita T, Hirose T, Kawamori R, et al (2009) Effects of exendin-4 on glucose tolerance, insulin secretion, and beta-cell proliferation depend on treatment dose, treatment duration and meal contents. Biochem Biophys Res Comm 390: 809–814. 19836346 10.1016/j.bbrc.2009.10.054

[69] Edwards CMB, Stanley SA, Davis R, Brynes AE, Frost GS, et al (2001) Exendin-4 reduces fasting and postprandial glucose and decreases energy intake in healthy volunteers. Am J Physiol Endocrinol Metab 281: E155–161. 11404233 10.1152/ajpendo.2001.281.1.E155

[70] DeFronzo RA, Okerson T, Viswanathan P, Guan X, Holcombe JH, et al (2008) Effects of exenatide versus sitagliptin on postprandial glucose, insulin and glucagon secretion, gastric emptying, and caloric intake: a randomized, cross-over study. Curr Med Res Opin 24: 2943–2952. 18786299 10.1185/03007990802418851

[71] Linnebjerg H, Park S, Kothare PA, Trautmann ME, Mace K, et al (2008) Effect of exenatide on gastric emptying and relationship to postprandial glycemia in type 2 diabetes. Regulatory Peptides DOI: 10.1016/j.regpep.2008.07.003. 10.1016/j.regpep.2008.07.003 18675854

[72] Sano H, Takebayashi A, Kodama Y, Nakamura K, Ito H, et al (1999) Effects of feed restriction and cold exposure on glucose metabolism in response to feeding and insulin in sheep. J Anim Sci 77: 2564–2573. 10492466 10.2527/1999.7792564x

[73] Katsumata M, Burton KA, Li J, Dauncey MJ (1999) Suboptimal energy balance selectively up-regulates muscle GLUT gene expression but reduces insulin-dependent glucose uptake during postnatal development. FASEB J 13: 1405–1413. 10428764 10.1096/fasebj.13.11.1405

[74] Kastin AJ, Akerstrom V (2003) Entry of exendin-4 into brain is rapid but may be limited at high doses. Int J Obesity Rel Metab Disorders 27: 313. 10.1038/sj.ijo.0802206 12629557

[75] Kwon DY, Kim YS, Ahn IS, Kim DS, Kang S, et al (2009) Exendin-4 potentiates insulinotropic action partly via increasing β-cell proliferation and neogenesis and decreasing apoptosis in association with the attenuation of endoplasmic reticulum stress in islets of diabetic rats. Journal of Pharmacological Sciences 111: 361–371. 20019445 10.1254/jphs.09178fp

[76] Fehse F, Trautmann M, Holst JJ, Halseth AE, Nanayakkara N, et al (2005) Exenatide augments first- and second-phase insulin secretion in response to intravenous glucose in subjects with type 2 diabetes. Journal of Clinical Endocrinology and Metabolism 90: 5991–5997. 16144950 10.1210/jc.2005-1093

[77] Young AA, Gedulin BR, Bhavsar S, Bodkin N, Jodka C, et al (1999) Glucose-lowering and insulin-sensitizing actions of exendin-4: studies in obese diabetic (ob/ob, db/db) mice, diabetic fatty Zucker rats, and diabetic rhesus monkeys (Macaca mulatta). Diabetes 48: 1026–1034. 10331407 10.2337/diabetes.48.5.1026

[78] Gedulin BR, Nikoulina SE, Smith PA, Gedulin G, Nielsen LL, et al (2005) Exenatide (Exendin-4) improves insulin sensitivity and β-cell mass in insulin-resistant obese *fa*/*fa* Zucker rats independent of glycemia and body weight. Endocrinology 146: 2069–2076. 15618356 10.1210/en.2004-1349

[79] Cersosimo E, Gastaldelli A, Cervera A, Wajcberg E, Sriwijilkamol A, et al (2011) Effect of exenatide on splanchnic and peripheral glucose metabolism in type 2 diabetic subjects. Journal of Clinical Endocrinology & Metabolism 96: 1763–1770. 21411546 10.1210/jc.2010-2146

[80] Belfiore F, Iannello S (1998) Insulin resistance in obesity: Metabolic mechanisms and measurement methods. Mol Genetics Metab 65: 121–128. 10.1006/mgme.1998.2727 9787104

[81] Summermatter S, Marcelino H, Arsenijevic D, Buchala A, Aprikian O, et al (2009) Adipose tissue plasticity during catch-up fat driven by thrifty metabolism. Diabetes 58: 2228–2237. 19602538 10.2337/db08-1793 PMC2750217

[82] Forsén T, Eriksson J, Tuomilehto J, Reunanen A, Osmond C, et al (2000) The fetal and childhood growth of persons who develop type 2 diabetes. Ann Internal Med 133: 176–182. 10906831 10.7326/0003-4819-133-3-200008010-00008

[83] Ong KKL, Ahmed ML, Emmett PM, Preece MA, Dunger DB (2000) Association between postnatal catch-up growth and obesity in childhood: prospective cohort study. BMJ 320: 967–971. 10753147 10.1136/bmj.320.7240.967 PMC27335

[84] De Blasio MJ, Gatford KL, Robinson JS, Owens JA (2006) Placental restriction alters circulating thyroid hormone in the young lamb postnatally. American Journal of Physiology 291: R1016–R1024. 16627695 10.1152/ajpregu.00103.2006

[85] De Blasio MJ, Gatford KL, Robinson JS, Owens JA (2007) Placental restriction of fetal growth reduces size at birth and alters postnatal growth, feeding activity and adiposity in the young lamb. American Journal of Physiology 292: R875–R886. 17023666 10.1152/ajpregu.00430.2006

