# Correction: Neonatal Exendin-4 Reduces Growth, Fat Deposition and Glucose Tolerance during Treatment in the Intrauterine Growth-Restricted Lamb

**DOI:** 10.1371/journal.pone.0095944

**Published:** 2014-04-25

**Authors:** The PLOS ONE Staff

Figure 3 is missing the x-axis label and the indicators of significance. The authors have provided a corrected version of Figure 3 here.

**Figure 3:**
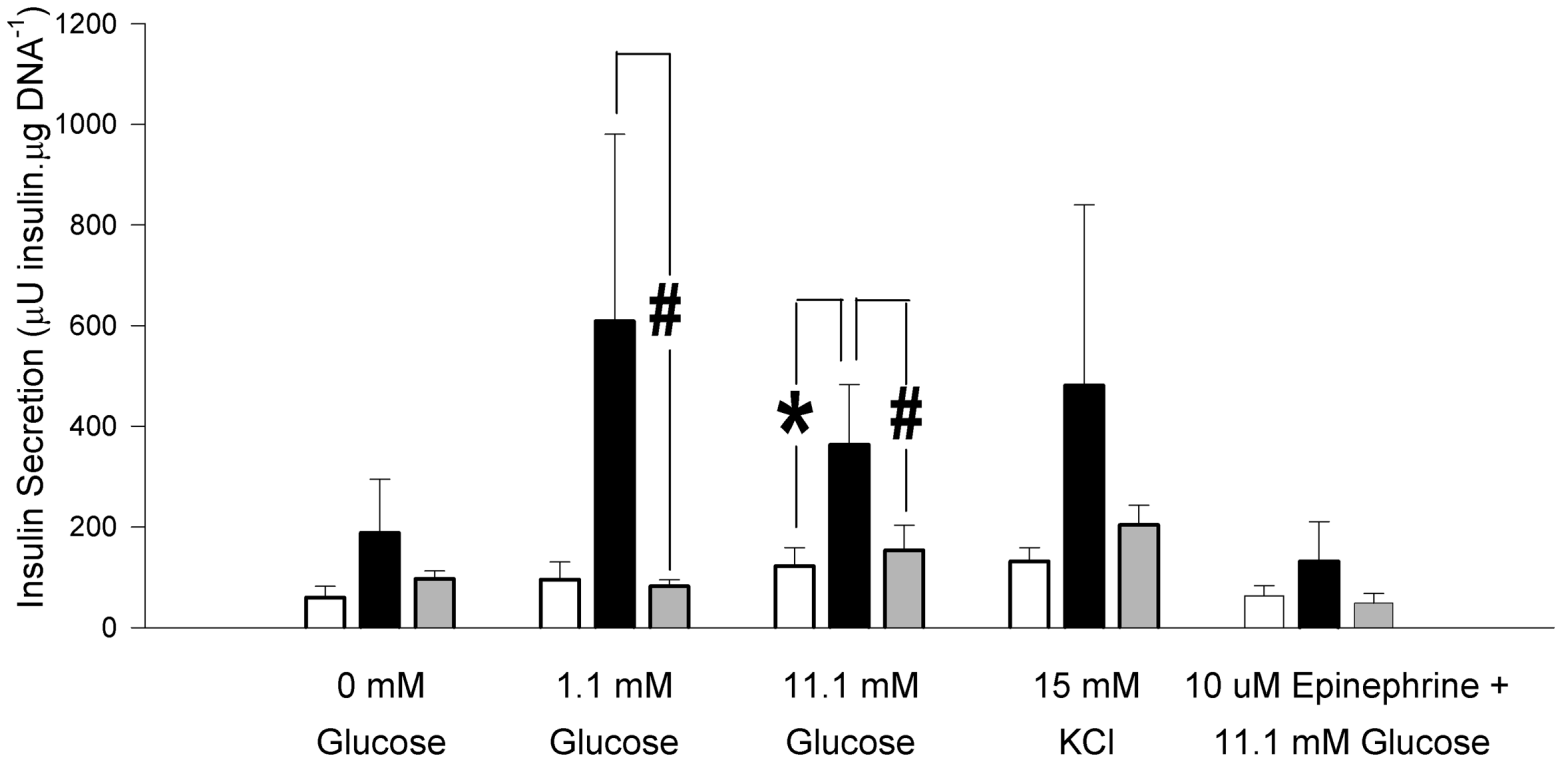
**Effect of IUGR and neonatal exendin-4 treatment on in vitro insulin secretion from isolated islets in response to glucose and potassium chloride.** CON (white bar, n  =  5), IUGR+Veh (black bar, n  =  5) and IUGR+Ex-4 (gray bar, n  =  6). Data are means ± SEM. Specific contrasts: ^*^ P<0.05, ^#^ P<0.10.
